# A Bioengineered Nisin Derivative to Control Biofilms of *Staphylococcus pseudintermedius*

**DOI:** 10.1371/journal.pone.0119684

**Published:** 2015-03-19

**Authors:** Des Field, Noémie Gaudin, Francy Lyons, Paula M. O'Connor, Paul D. Cotter, Colin Hill, R. Paul Ross

**Affiliations:** 1 School of Microbiology, University College Cork, Cork, Ireland; 2 Teagasc Food Research Centre, Moorepark, Fermoy, County Cork, Ireland; 3 Alimentary Pharmabiotic Centre, University College Cork, Cork, Ireland; The University of Nevada - Reno, UNITED STATES

## Abstract

Antibiotic resistance and the shortage of novel antimicrobials are among the biggest challenges facing society. One of the major factors contributing to resistance is the use of frontline clinical antibiotics in veterinary practice. In order to properly manage dwindling antibiotic resources, we must identify antimicrobials that are specifically targeted to veterinary applications. Nisin is a member of the lantibiotic family of antimicrobial peptides that exhibit potent antibacterial activity against many gram-positive bacteria, including human and animal pathogens such as *Staphylococcus*, *Bacillus*, *Listeria*, and *Clostridium*. Although not currently used in human medicine, nisin is already employed commercially as an anti-mastitis product in the veterinary field. Recently we have used bioengineering strategies to enhance the activity of nisin against several high profile targets, including multi-drug resistant clinical pathogens such as methicillin-resistant *Staphylococcus aureus* (MRSA) and vancomycin-resistant enterococci (VRE) and also against staphylococci and streptococci associated with bovine mastitis. However, newly emerging pathogens such as methicillin resistant *Staphylococcus pseudintermedius* (MRSP) pose a significant threat in terms of veterinary health and as a reservoir for antibiotic resistance determinants. In this study we created a nisin derivative with enhanced antimicrobial activity against *S*. *pseudintermedius*. In addition, the novel nisin derivative exhibits an enhanced ability to impair biofilm formation and to reduce the density of established biofilms. The activities of this peptide represent a significant improvement over that of the wild-type nisin peptide and merit further investigation with a view to their use to treat *S*. *pseudintermedius* infections.

## Introduction

**Fig 1:**
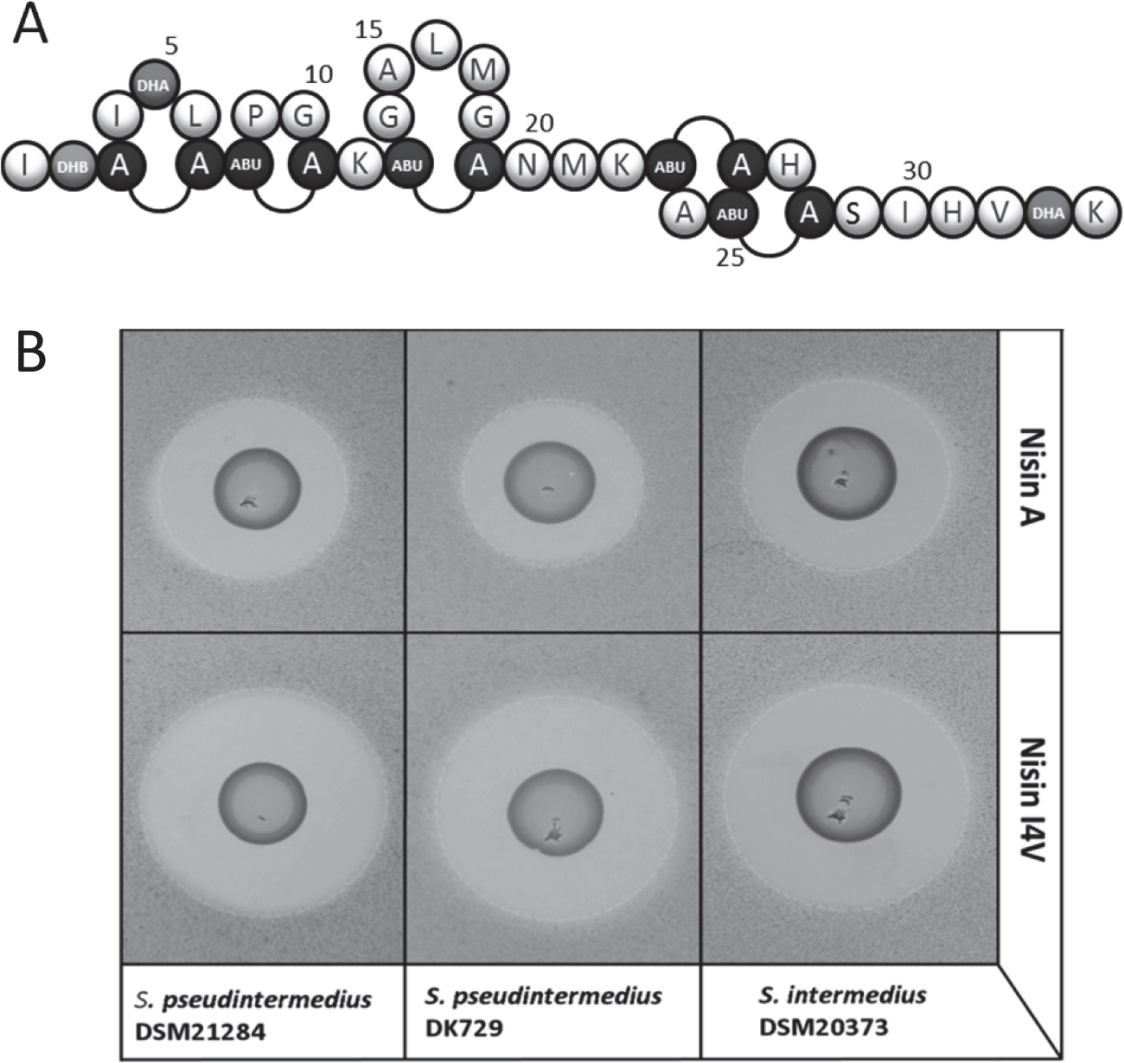
Structure of nisin A and deferred antagonism assays of nisin A and nisin I4V. (A) Residues are represented in the single letter code. Post translational modifications are indicated as follows, Dha: dehydroalanine, Dhb: dehydrobutyrine, Abu: 2-aminobutyric acid, A-A: lanthionine, Abu-A: 3-methyllanthionine. (B) Growth inhibition of *S*. *intermedius* DSM 20373, *S*. *pseudintermedius* DK729 and *S*. *pseudintermedius* DSM21284 by the nisin A producing strain *L*. *lactis* NZ9800 pDF05 (pCI372-*nis*A) and the nisin derivative I4V producing strain *L*. *lactis* NZ9800 pDF12 (*nis*A-I4V).

The diminished capacity of currently available antibiotics to control pathogenic bacteria is a major cause for concern. Against this backdrop, methicillin-resistant *Staphylococcus pseudintermedius* (MRSP) has emerged over the last decade as a critically important, opportunistic canine pathogen responsible for skin, soft tissue, and surgical site infections [1]. Although frequently detected in dogs, MRSP has also been isolated from several other host species including cats, horses, donkeys and birds [2]. Worryingly, MRSP has implications for public health as transmission between humans and their pets can occur *via* direct and indirect contact and infections in humans have been described [3]. MRSP can form biofilms [4,5]; complex, sessile communities of bacteria embedded in an organic polymer matrix [6]. Biofilm formation is now recognized as an important virulence factor in several *Staphylococcus* spp. [7], providing the bacteria with chemical and physical protection from the host immune response and the effects of antimicrobials [8]. In addition to methicillin resistance and biofilm formation, the acquisition of other resistance genes and resistance-mediating mutations in some MRSP isolates renders these strains resistant to the majority of antimicrobial agents utilized in veterinary medicine [9]. MRSP isolates are typically resistant to aminoglycosides, fluoroquinolones, macrolides, lincosamides, trimethoprim sulfamethoxazol and, in many cases, to tetracycline and chloramphenicol [9–11]. Thus new alternatives to conventional antibiotic therapies are urgently needed. One group of compounds with enormous potential for therapeutic use is the lantibiotic class of bacteriocins (bacterially derived antimicrobial peptides) [12,13]. Lantibiotics are gene-encoded, ribosomally-synthesized peptides that are characterised by the presence of unusual amino acids including lanthionine and/or methyllanthionine [14–16]. The most intensively studied lantibiotic is nisin (Fig. 1A). Produced by *Lactococcus lactis*, nisin exhibits antibacterial activity against a wide range of Gram-positive bacteria, including foodborne pathogens such as staphylococci, bacilli and clostridia. Nisin is used as a food preservative in over 50 countries and has been approved in the EU (as additive E234) and by the US Food and Drug Administration (FDA) [17]. In addition, both nisin A (and its natural variant nisin Z) are effective against the Gram positive pathogens responsible for bovine mastitis and have been incorporated into a number of products (such as Wipe Out and Mast Out) dedicated to controlling or treating such infections [12,18–20]. Additionally, Bayer have recently released Preva medicated wipes containing 25μg/ml nisin for topical use on dogs, cats, and horses with dermatological conditions associated with bacterial infections or general cleansing (www.animalhealth.bayerhealthcare.com). Notably, in addition to being effective against planktonic cells of multi-drug resistant staphylococci [21,22], nisin has also demonstrated efficacy against biofilms [22–24]. This activity can be further enhanced by taking advantage of the fact that nisin functions synergistically with several conventional antibiotics against biofilms of MRSA [25].

The mode of action of nisin and several other lantibiotics has been elucidated, revealing that membrane-bound peptidoglycan precursor lipid II acts as an initial docking molecule. In the case of nisin, this results in both the inhibition of cell wall biosynthesis and the disruption of the cell membrane due to pore formation [26,27]. As a consequence of these two distinct and co-operative mechanisms, microbes have been unable to develop any significant resistance to nisin outside of the laboratory despite its widespread use in the food industry [14]. The ribosomal origin of lantibiotics facilitates approaches that can alter the structure of the mature peptides in a more precise fashion than is possible for classical (non-ribosomal) antimicrobials, and consequently enables the creation of lantibiotic variants with altered biological, chemical and physical properties. Bioengineering strategies have been rewarding with respect to the introduction of mutations that have a beneficial impact on the physico-chemical properties of nisin, including better solubility and improved stability [28,29] as well as an enhanced ability to diffuse through complex polymers [30]. With respect to increasing antimicrobial activity, this was initially achieved through the creation of bioengineered nisin derivatives with superior antimicrobial activity against some non-pathogenic targets [31,32]. Notably, nisin Z N20K and M21K (N20K denotes a change from Asn to Lysine at position 20 of the propeptide) were the first bioengineered nisin derivatives to show enhanced activity against pathogenic bacteria, namely *Shigella*, *Pseudomonas* and *Salmonella* species [29]. The first nisin derivatives with improved activity against Gram-positive pathogens were nisin A N20P, M21V, K22S and K22T [33]. Nisin A M21V has since been designated as nisin V and has also been found to exhibit enhanced activity against a wide range of targets, including numerous drug resistant strains [34]. A more comprehensive bioengineering of the hinge region (residues 20–22) revealed the benefits of incorporating small chiral amino acids leading to the rational design of nisin derivatives with enhanced properties [35]. Outside of the hinge region, several nisin A derivatives were described that displayed increased potency against a range of Gram-positive targets, with S29G and S29A representing the first nisin derivatives which display enhanced activity against both Gram-positive and Gram-negative bacteria [36]. Finally, derivatives bioengineered at lysine 12 (K12), located in a flexible region located between rings B and C of the peptide also displayed enhanced activity [37]. Collectively, these studies demonstrate that bioengineering can both improve the activity of nisin against sensitive cells as well as alter its target spectrum. In this study, we screen a bank of nisin derivatives to identify novel peptides that exhibit enhanced potency against an MRSP target strain, *S*. *pseudintermedius* DK729. A novel variant was identified with improved potency against strains of *S*. *pseudintermedius* in deferred antagonism and minimum inhibitory concentration assays. Importantly, the nisin derivative was also more effective in preventing biofilm formation, and in reducing the biofilm mass formed on microtiter plates. To our knowledge, this is the first report of a bioengineered lantibiotic peptide with enhanced efficacy against this pathogen or against bacterial biofilms.

## Materials and Methods

### Bacterial Strains and Growth Conditions

*L*. *lactis* strains were grown in M17 broth supplemented with 0.5% glucose (GM17) or GM17 agar at 30°C. *E*. *coli* was grown in Luria-Bertani broth with vigorous shaking or agar at 37°C. *Staphylococcus*, *Bacillus* and *Streptococcus* strains were grown in Brain Heart Infusion (BHI) or BHI agar at 37°C. Antibiotics were used where indicated at the following concentrations: Chloramphenicol at 10 and 20 μg ml^-1^, respectively for *L*. *lactis* and *E*. *coli*.

### Creation and screening a bank of nisin derivatives

Mutagenesis of the *nis*A gene was carried out as described previously [33]. Briefly, saturation mutagenesis was carried out using pDF05 (pCI372-*nisA*) as template and using oligonucleotides as listed in (Table 1) containing an NNK codon in place of each native codon. PCR amplification was performed in a 50 μl reaction containing approximately 0.5 ng of target DNA (pDF05), 1 unit Phusion High-Fidelity DNA polymerase (Finnzymes, Finland), 1 mM dNTPs and 500 ng each of the appropriate forward and reverse oligonucleotide. The reaction was pre-heated at 98°C for 2 min, and then incubated for 29 cycles at 98°C for 30 s, 55°C for 15 s and 72°C for 3 min 30 s, and then finished by incubating at 72°C for 3 min 30 s. Amplified products were treated with Dpn1 (Stratagene) for 60 min at 37°C to digest template DNA and purified using the QIAquick PCR purification kit. Following transformation of *E*. *coli* Top 10 cells plasmid DNA was isolated and sequenced using primers pCI372FOR and pCI372REV (Table 1) to verify that mutagenesis had taken place. Approximately 150 transformants were chosen at random for each position and inoculated into 96-well plates containing GM17 chloramphenicol, incubated overnight and stored at −20°C after addition of 80% glycerol. Deferred antagonism assays were performed by replicating strains on GM17 agar plates and allowing them to grow overnight before overlaying with BHI agar (0.75% w/v agar) seeded with the appropriate indicator strain.

**Table 1:** Oligonucleotides utilised in this study.

Primer name	Sequence
NisI1degFOR	5’ ATT ACA AGT **NNK** TCG CTA TGT ACA CCC GGT TGT AAA ACA 3’
NisI1degREV	5’ ACA TAG CGA **MNN** ACT TGT AAT GCG GGT TGA TGC ACC TGA 3’
NisT2degFOR	5’ CCA CGC ATT **NNK** AGT ATT TCG CTA TGT ACA CCC GGT TGT 3’
NisT2degREV	5’ CGA AAT ACT **MNN** AAT GCG GGT TGA TGC ACC TGA ATC TTT 3’
NisI4degFOR	5’ ATT ACA AGT **NNK** TCG CTA TGT ACA CCC GGT TGT AAA ACA 3’
NisI4degREV	5’ ACA TAG CGA **MNN** ACT TGT AAT GCG TGG TGA TGC ACC TGA 3’
NisS5degFOR	5’ ACA AGT ATT **NNK** CTA TGT ACA CCC GGT TGT AAA ACA GGA 3’
NisS5degREV	5’ TGT ACA TAG **MNN** AAT ACT TGT AAT GCG TGG TGA TGC ACC 3’
NisL6degFOR	5’ AGT ATT TCG **NNK** TGT ACA CCC GGT TGT AAA ACA GGA GCT 3’
NisL6degREV	5’ GGG TGT ACA **MNN** CGA AAT ACT TGT AAT GCG TGG TGA TGC 3’
NisP9degFOR	5’ CTA TGT ACA **NNK** GGT TGT AAA ACA GGA GCT CTG ATG GGT 3’
NisP9degREV	5’ TTT ACA ACC **MNN** TGT ACA TAG CGA AAT ACT TGT AAT GCG 3’
NisG10degFOR	5’ TGT ACA CCC **NNK** TGT AAA ACA GGA GCT CTG ATG GGT TGT 3’
NisG10degREV	5’ TGT TTT ACA **MNN** GGG TGT ACA TAG CGA AAT ACT TGT AAT 3’
NisG14degFOR	5’ TGT AAA ACA **NNK** GCT CTG ATG GGT TGT AAC ATG AAA ACA 3’
NisG14degREV	5’ CAT CAG AGC **MNN** TGT TTT ACA ACC GGG TGT ACA TAG CGA 3’
NisA15degFOR	5’ AAA ACA GGA **NNK** CTG ATG GGT TGT AAC ATG AAA ACA GCA 3’
NisA15degREV	5’ ACC CAT CAG **MNN** TCC TGT TTT ACA ACC GGG TGT ACA TAG 3’
NisL16degFOR	5’ ACA GGA GCT **NNK** ATG GGT TGT AAC ATG AAA ACA GCA ACT 3’
NisL16degREV	5’ ACA ACC CAT **MNN** CGA TCC TGT TTT ACA ACC GGG TGT ACA 3’
NisM17degFOR	5’ GGA GCT CTG **NNK** GGT TGT AAC ATG AAA ACA GCA ACT TGT3’
NisM17degREV	5’ GTT ACA ACC **MNN** CAG AGC TCC TGT TTT ACA ACC GGG TGT 3’
NisG18degFOR	5’ GCT CTG ATG **NNK** TGT AAC ATG AAA ACA GCA ACT TGT CAT 3’
NisG18degREV	5’ CAT GTT ACA **MNN** CAT CAG AGC TCC TGT TTT ACA ACC GGG 3’
NisA24degFOR	5’ ATG AAA ACA **NNK** ACT TGT CAT TGT AGT ATT CAC GTA AGC 3’
NisA24degREV	5’ ATG ACA AGT **MNN** TGT TTT CAT GTT ACA ACC CAT CAG AGC 3’
NisH27degFOR	5’ GCA ACT TGT **NNK** TGT AGT ATT CAC GTA AGC AAA TAA TCT 3’
NisH27degREV	5’AAT ACT ACA **MNN** ACA AGT TGC TGT TTT CAT GTT ACA ACC 3’
NisI30degFOR	5’ CAT TGT AGT **NNK** CAC GTA AGC AAA TAA TCT AGA GTCG AC 3’
NisI30degREV	5’ TTT GCT TAC GTG **MNN** ACT ACA ATG ACA AGT TGC TGT TTT 3’
NisH31degFOR	5’ TGT AGT ATT **NNK** GTA AGC AAA TAA TCT AGA GTC GAC CTG 3’
NisH31degREV	5’ TTT GCT TAC **MNN** AAT ACT ACA ATG ACA AGT TGC TGT TTT 3’
NisV32degFOR	5’ AGT ATT CAC **NNK** AGC AAA TAA TCT AGA GTC GAC CTG CAG 3’
NisV32degREV	5’ TTA TTT GCT **MNN** GTG AAT ACT ACA ATG ACA AGT TGC TGT 3’
NisS33degFOR	5’ ATT CAC GTA **NNK** AAA TAA TCT AGA GTC GAC CTG CAG CAA 3’
NisS33degREV	5’AGA TTA TTT **MNN** TAC GTG AAT ACT ACA ATG ACA AGT TGC 3’
NisK34degFOR	5’ CAC GTA AGC **NNK** TAA TCT AGA GTC GAC CTG CAG CAA TGG 3’
NisK34degREV	5’ TCT AGA TTA **MNN** GCT TAC GTG AAT ACT ACA ATG ACA AGT 3’
pCI372FOR	5’- CGGGAAGCTAGAGTAAGTAG -3'
pCI372Rev	5’- ACCTCTCGGTTATGAGTTAG -3’

Emboldened sequences represent degenerate codons (N = A+C+G+T, K = G+T, M = A+C). Underlined sequence corresponds to plasmid (pCI372) DNA.

### Mass Spectrometry

For Colony Mass Spectrometry (CMS) bacteria were collected with sterile plastic loops and mixed with 50 μl of 70% isopropanol adjusted to pH 2 with HCl. The suspension was vortexed, the cells spun down in a benchtop centrifuge at 14,000 r.p.m. for 2 min, and the supernatant was removed for analysis. Mass Spectrometry in all cases was performed with an Axima CFR plus MALDI TOF mass spectrometer (Shimadzu Biotech, Manchester, UK). A 0.5μl aliquot of matrix solution (alpha-cyano-4-hydroxy cinnamic acid (CHCA), 10 mg ml^-1^ in 50% acetonitrile-0.1% (v/v) trifluoroacetic acid) was placed onto the target and left for 1–2 min before being removed. The residual solution was then air dried and the sample solution (resuspended lyophilised powder or CMS supernatant) was positioned onto the precoated sample spot. Matrix solution (0.5μl) was added to the sample and allowed to air-dry. The sample was subsequently analysed in positive-ion reflectron mode.

### Nisin purification

2 litres of Tryptone Yeast (TY) broth were incubated for 20 hours with 1% inoculum of an overnight culture of producing strain. This culture was centrifuged for 20 minutes @ 7000rpm. The supernatant was decanted and passed through 60 g of pre equilibrated Amberlite XAD16 beads (Sigma-Aldrich). The beads were washed with 500 ml 30% ethanol and eluted with 500 ml 70% isopropanol (IPA) (Fisher) 0.1% trifluoroacetic acid (TFA) (Sigma-Aldrich). Concomitantly, the cell pellets were resuspended in 300 ml of 70% IPA 0.1%TFA and stirred at room temperature for 3 hours followed by centrifugation. This cell supernatant was combined with that referred to above and concentrated through rotary-evaporation (Buchi, Switzerland) to approximately 250 ml. Following pH adjustment to 4.0 further concentration was achieved through the use of a Phenomenex SPE C-18 column to a final volume of 60 ml. 8 ml of this sample was concentrated, through rotary evaporation, to 2 ml and purified through HPLC using a Phenomenex C12 Reverse-Phase (RP) HPLC column (Jupiter 4 μ proteo 90 Å, 250 X 10.0 mm, 4 μm). To facilitate this, a gradient of 30–50% acetonitrile (Fisher) containing 0.1% TFA was developed. The relevant fractions were collected and pooled, subjected to rotary-evaporation to remove acetonitrile and freeze-dried (LABCONCO). The purified peptides were subjected to MALDI-ToF Mass Spectrometric analysis to confirm their purity before use.

### Minimum Inhibitory Concentration assays

Minimum inhibitory concentration determinations were carried out in triplicate in 96 well microtitre plates. 96 well microtitre plates were pre-treated with bovine serum albumin (BSA) prior to addition of the peptides. Briefly, to each well of the microtitre plate 200 μL of phosphate buffered saline (PBS) containing 1% (w/v) bovine serum albumin (PBS/BSA) was added and incubated at 37°C for 30 min. The wells were washed with 200 μL PBS and allowed to dry. Target strains were grown overnight in the appropriate conditions and medium, subcultured into fresh broth and allowed to grow to an OD_600_ of ∼0.5, diluted to a final concentration of 10^5^ cfu ml^−1^ in a volume of 0.2 ml. The lyophilised peptides were resuspended in 0.005% acetic acid to a stock concentration of 30 μM. Wild type nisin and nisin I4V mutant peptides were adjusted to a 7.5 or 5.0 μM or 500 nM starting concentration and 2-fold serial dilutions of each peptide were made in 96 well plates for a total of 12 dilutions. The target strain was then added and after incubation for 16 h at 30°C or 37°C the MIC was read as the lowest peptide concentration causing inhibition of visible growth.

### Growth curve experiments

For growth experiments, overnight cultures were transferred (10^7^ cfu ml^−1^ in a volume of 1.0 ml.) into BHI supplemented with the relevant concentration of wild-type and I4V peptides, and subsequently 0.2mls was transferred to 96 well microtitre plates (Sarstedt). Cell growth was measured spectrophotometrically over 24-h periods by using a SpectraMax spectrophotometer (Molecular Devices, Sunnyvale, Calif.).

### Biofilm formation

Static microtitre plate assays based on a previous study [38], but with modifications to optimize the assay, were used to investigate the biofilm formation and nisin treatments. TSB (Merck) broth supplemented with 1% D-(+)-glucose (Sigma Aldrich) (TSBg) was used in these assays which aids in biofilm formation. Briefly, a 1: 100 dilution was performed by adding 2 μl of log phase cells (10^7^ CFU ml^−1^ of each culture) to 198 μl of TSBg in wells of a sterile 96-well microtitre plate (Sarstedt, Leicester, UK), giving a starting inoculum of 10^5^ CFU ml^−1^; 200 μl of TSBg was added to a set of wells as a negative control. All wells were seeded in triplicate. Microtitre plates were then incubated at 37°C for 48 h to allow biofilm formation to occur. Washing (PBS) and staining of wells (0.05% crystal violet) was carried out as described previously [39].

### Biofilm prevention with nisin peptides

The ability of nisin and the nisin mutant I4V to prevent biofilm formation was carried out as described above with the following modifications. At the beginning of the assay, nisin peptides were added to the microtitre plate wells at 1X 1/2X, 1/4X, 1/8X, 1/16X MIC in TSBg and incubated at 37°C for 24 hrs. *S*. *pseudintermedius* cells alone were inoculated into a third set of wells as untreated controls. The plates were removed and gently washed once with PBS and stained with 0.05% crystal violet as described previously, and optical density readings were taken at 595 nm (OD_595_) to determine the final biofilm biomass.

### Biofilm treatment with purified nisin A and nisin derivative I4V peptides

Biofilm formation was carried out as described earlier. After biofilms were established and washed once with phosphate buffered saline (PBS), nisin peptides were added to the microtitre plate wells at 1X, 2X, 4X, 8X and 16X the relevant MIC as previously determined. All wells were seeded in triplicate. Following incubation for 24 h, at 37°C, the plates were removed and gently washed once with PBS and stained with 0.05% crystal violet as described previously, and optical density readings were taken at 595 nm (OD_595_) to determine the final biofilm biomass. Alternatively, following incubation for 24 h, at 37°C, the biofilms exposed to peptides at 16X MIC were gently washed once with PBS then 100 μL of a solution containing 500 mg XTT/L (2,3-bis[2-methyloxy-4-nitro-5-sulfophenyl]-2H-tetrazolium-5-carboxanilide) (Sigma) and 10mM menadione (Sigma) was added to each well. Microtitre plates were incubated for 2 h at 37°C in the dark. Absorbance was measured at 490nm using a microtiter plate reader (Molecular Devices Spectramax M3, Sunnyvale CA, USA). Data obtained in triplicate were calculated and expressed as the mean ± standard deviations.

## Results

### Identification of nisin derivatives with enhanced bioactivity against *S*. *pseudintermedius* and *S*. *intermedius*

A site-saturation mutagenesis-based strategy was used to generate a bank of strains producing bioengineered nisin derivatives in which 19 residues (I1, T2, I4, S5, L6, P9, G10, G14, A15, L16, M17, G18, A24, H27, I30, H31, V32, S33 and K34) were each randomised to potentially all other natural amino acids. These 19 residues include all those not involved in lanthionine ring formation, and those which had previously been the focus of bioengineering based investigations in our laboratory (K12, N20, M21, K22 and S29). The resultant bank of approximately 3,000 individual producers was screened using deferred antagonism agar diffusion assays to identify those with display enhanced bioactivity (which reflects a combination of production and specific activity). Candidates were distinguished by zones of clearing that were greater than those generated by the nisin A producing control against the target *S*. *pseudintermedius* DK729. From this screen 47 potentially enhanced producers were selected for further investigation. Mass spectrometric analysis of the peptide produced by each strain established that these corresponded to 10 unique mutants (data not shown). However, the variant with an isoleucine to valine substitution located within ring A at position 4 (I4V) consistently exhibited improved bioactivity in deferred antagonism assays against the *S*. *pseudintermedius* (DK729, DSM21284) and *S*. *intermedius* (DSM20373) strains utilized in this study (Fig. 1B; Table 2). On this basis, the nisin I4V derivative was selected for purification and specific activity assays. The nine other enhanced derivatives will be the subject of future investigations.

**Table 2:** Deferred antagonism assays of *L. lactis* NZ9800 strains producing nisin A (wild type control) and the nisin derivative I4V against representative strains of *S. pseudintermedius* and *S. intermedius*.

Strain	Nisin A	Nisin I4V	P-value
	mm	mm	
*S. pseudintermedius* DK729	16.90 ± 0.48	**17.96 ± 0.50**	0.009
*S. pseudintermedius* DSM21284	16.88 ± 0.29	**18.06 ± 0.14**	0.008
*S. intermedius* DSM 20373	16.84 ± 0.27	**18.27 ± 0.07**	0.039

Values are the mean of triplicate deferred antagonism assays and represent zone of inhibition (diameter of zone). All values in bold reached statistical significance compared to nisin control (Student’s t-test: P < 0.05).

### MIC-based investigations demonstrate enhanced specific activity of nisin I4V

Although deferred antagonism agar diffusion assays can rapidly assess the bioactivity of a producer strain, differences in zone size can result from altered diffusion rates in agar, or from increased levels of production, or from changes in specific activity. To confirm that the enhanced activity of variant I4V was due to increased specific activity, the activity of the purified peptide was assessed using classical broth-based minimum inhibitory concentration (MIC) determination assays. Following high performance liquid chromatography (HPLC) and freeze-drying to obtain purified peptide, MIC assays were carried out using equimolar concentrations of nisin A and nisin A I4V against a range of Gram positive targets including *S*. *pseudintermedius* DK729, *S*. *pseudintermedius* DSM21284, *S*. *intermedius* DSM20373, *S*. *aureus* DPC5243, *S*. *aureus* RF122, *S*. *uberis* ATCC, *B*. *cereus* DPC6087 and *Lactococcus lactis* spp. *lactis* HP. The MIC was determined to be the lowest concentration of peptide that resulted in the absence of visible growth of the target strain after 16 hours at 37°C. This method established an MIC of 3.0 mg/L for nisin A against *S*. *intermedius* DSM20373 (Table 3). This is within the MIC range described in a previous study carried out with nisin (MIC90 of 3.2 mg/L) against a selection of methicillin resistant *staphylococci* (*S*. *aureus*, *S*. *intermedius and S*. *schleiferi*) [40]. In contrast, the MIC of nisin A I4V was determined to be 1.5 mg/L (0.468 μM), a two-fold increase in specific activity. MIC determinations established that the MIC of nisin A for *S*. *pseudintermedius* DK729 and *S*. *pseudintermedius* DSM21284 was 2 and 1 mg/L (0.625 and 0.313μM), respectively. In contrast, the MIC of nisin I4V was determined to be 1 and 0.25 mg/L (0.313 and 0.075 μM) against the same targets, reflecting a 2 and 4 fold increase in specific activity (Table 3). To determine if the enhanced activity of I4V relative to nisin A is target specific MICs were assessed against a number of other targets. *S*. *aureus* DPC5243 and *S*. *aureus* RF122 were chosen, both of which were originally associated with bovine mastitis [41,42]. Against DPC5243, the I4V derivative again displayed a two-fold decrease in potency as evident by MIC values of 1.50 mg/L and 0.75 mg/L (0.468μM and 0.234μM), respectively. However, no difference in specific activity was observed against *S*. *aureus* RF122, with an MIC value of 1.5 mg/L (0.468 μM) for both parent and variant nisin. The I4V derivative displayed enhanced efficacy (12.57 mg/L) relative to nisin A (25 mg/L) against the strain *S*. *uberis* ATCC 700407 (also associated with bovine-mastitis). However, no difference in MIC was observed in experiments with *B*. *cereus* DPC 6087 (6.28 mg/L) or *L*. *lactis* spp. *lactis* HP (0.2 mg/L). This variation in target specificity has previously been observed in the case of other nisin derivatives such as N20P, which displayed a two-fold increase in specific activity against *S*. *aureus* ST528 (MRSA) but was 75% less active against *Streptococcus agalactiae* ATCC13813 [33].

**Table 3:** Specific activity of nisin A and nisin I4V against a range of indicator organisms.

Strain	Nisin A	Nisin I4V	Fold difference
	mg/L (μM)	mg/L (μM)	
*S. pseudintermedius* DK729	2 (0.625)	**1 (0.313)**	2
*S. pseudintermedius* DSM21284	1 (0.313)	**0.25 (0.075)**	4
*S. intermedius* DSM 20373	3.0 (0.936)	**1.5 (0.468)**	2
*S. aureus* DPC 5243	0.75 (0.234)	1.50 (0.468)	-2
*S. aureus* RF122	1.5 (0.468)	1.5 (0.468)	0
*L. lactis* spp *lactis* HP	0.2 (0.062)	0.2 (0.062)	0
*S. uberis* ATCC	25 (7.5)	**12.57 (3.75)**	2
*B. cereus DPC 6087*	6.28 (1.875)	6.28 (1.875)	0

Results from minimum inhibitory concentration assays of purified nisin A and nisin I4V against various Gram-positive targets. Values given are identical results from three independent determinations. Fold Difference represents the improvement of I4V compared to nisin against the relevant indicator.

### Growth curve-based comparisons of the activity of nisin A and nisin I4V

**Fig 2:**
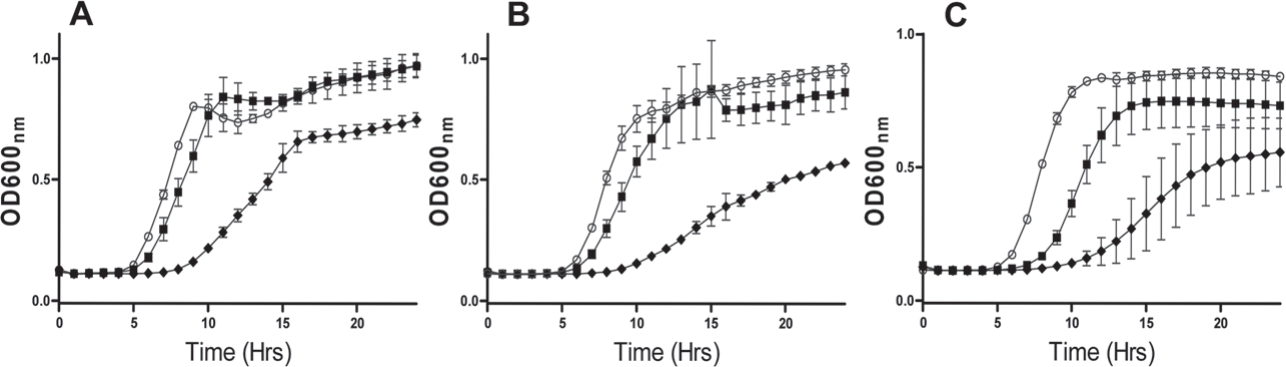
Growth curve analysis of strains in nisin A and nisin I4V peptides. (A) *S. pseudintermedius* DSM21284 in 0.26 mg/L of nisin A (closed square), I4V (closed diamond) and no peptide (open circle), and (B) *S. pseudintermedius* DK729 in 0.52 mg/L of Nisin A (closed square), I4V (closed diamond) and no peptide (open circle) and **(C)** *S. intermedius* DSM20373 in 0.52 mg/L of Nisin A (closed square), I4V (closed diamond) and no peptide (open circle).

Having established the enhanced specific activity of nisin I4V against the representative *S. pseudintermedius* and *S. intermedius* strains through end-point MIC assays, further analysis was carried out by means of growth curves in a bid to reveal more subtle details of the impact of nisin and nisin I4V peptides on bacterial viability. In each instance the results were consistent with the enhanced potency of I4V as revealed by MIC assays. For *S. pseudintermedius* DSM 21284 (1 X 10^7^ cfu/ml), nisin A caused a slight delay in growth relative to the non nisin-containing control at the concentration of peptide employed (0.26 mg/L) (Fig. 2A). Identical concentrations of nisin I4V resulted in a greatly extended lag time, highlighting its greater potency. Similar results were observed for *S. pseudintermedius* DK729 (Fig. 2B) and *S. intermedius* DSM 20373 (Fig. 2C), in that a sub-lethal concentration (0.52 mg/L) of the nisin A peptide brought about a slight delay in growth when compared to the non-peptide containing control, whereas the I4V peptide at the equivalent concentration extended the lag phase of growth by several hours.

### Investigation of the anti-biofilm activity of Nisin A and nisin I4V

**Fig 3:**
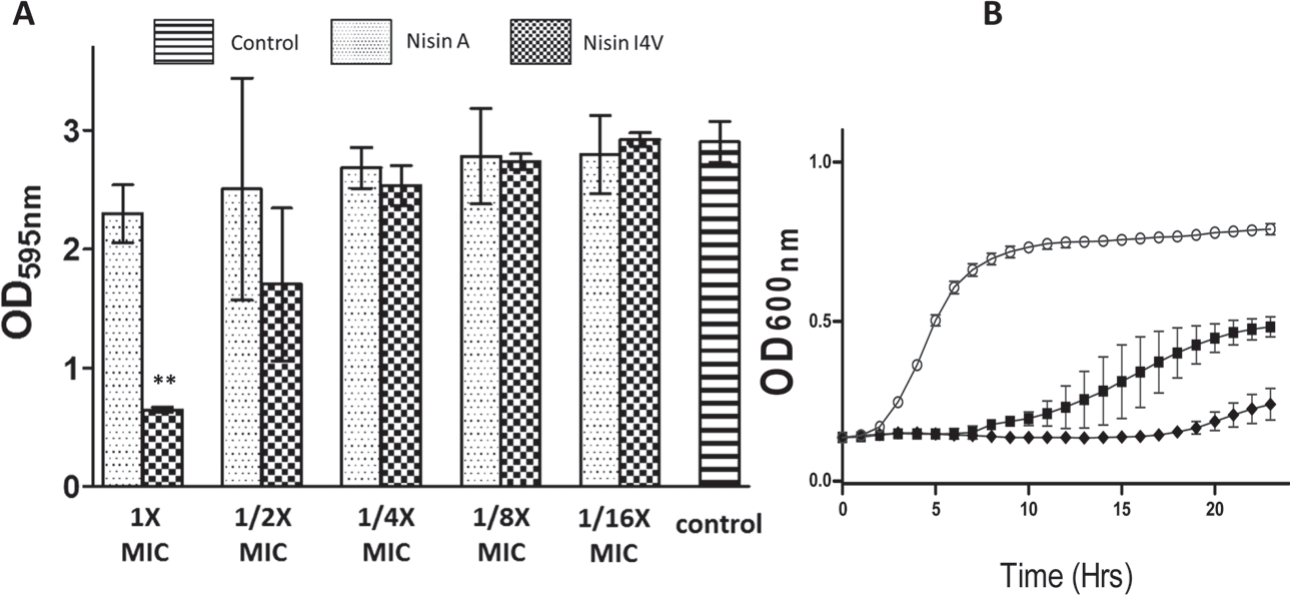
Inhibition of biofilm formation with nisin A and nisin I4V peptides. (A) Results of treatment of *S. pseudintermedius* DK729 with 1, 1/2, 1/4, 1/8 and 1/16X MIC of nisin A and nisin I4V peptides for 24 hrs prior to biofilm formation. The amount of biofilm was quantified by measuring the OD_595_ of crystal violet dissolved in acetic acid. The means and standard deviations of triplicate determinations are presented. Asterisks indicate statistically significant differences (Student’s t-test) between peptides used at similar concentration (** = *p* < 0.01) and (B) Growth curve analysis of strain *S. pseudintermedius* DK729 in 1X MIC peptides of nisin A (closed square), I4V (closed diamond) and no peptide (open circle).

Biofilm formation is now recognised as an important virulence factor among *Staphylococcus* species [7]. The ability to form a biofilm is only recently gaining attention in the case of *S. pseudintermedius* [4]. Indeed, in a study involving 140 *S. pseudintermedius* strains isolated from dogs, 96% were classified as strong or moderate biofilm producers [4]. The microtiter-plate test is one of the most frequently used techniques for quantifying biofilm formation [43,44]. Prior to initiating studies with nisin peptides, we evaluated the biofilm forming abilities of *S. pseudintermedius* DK729, *S. pseudintermedius* DSM21284 and *S. intermedius* DSM20373. All three strains formed biofilms (when grown in TSB supplemented with 1.0% glucose; TSBg) as determined using 96 well flat-bottomed polystyrene plates and analysed by crystal violet staining (data not shown). We employed the same methodology to study the ability of nisin A and nisin derivative I4V peptides to inhibit biofilm formation in the case of *S. pseudintermedius* DK729 as a representative strain and to observe the effect of increasing concentrations of nisin peptides on pre-formed biofilms of *S. pseudintermedius* DK729 and *S. pseudintermedius* DSM21284. For biofilm prevention studies, the MIC of nisin A against *S. pseudintermedius* DK729 was calculated to be at 0.625 μM (1X concentration). This concentration and several dilutions were added to the microtitre plate wells containing TSBg and the target strain before incubation at 37°C for 24 hrs. Following staining and optical density readings at 595 nm (OD_595_), a significant reduction in *S. pseudintermedius* DK729 biofilm mass was observed in wells containing 1X MIC of nisin I4V compared to those treated with 1X nisin A (Fig. 3A). When lower concentrations (1/2X, 1/4X, 1/8X and 1/16X) of the peptides were used, a similar biofilm density was observed for cells in the presence of nisin A, nisin I4V and the untreated control. Further analysis by way of growth curves was carried out at 0.625 μM (1X concentration) that revealed a greater inhibition on growth of *S. pseudintermedius* DK729 (Fig. 3B), indicating that at the concentration used the nisin I4V derivative is more effective in preventing biofilm formation than nisin A due to enhanced growth inhibition.

**Fig 4:**
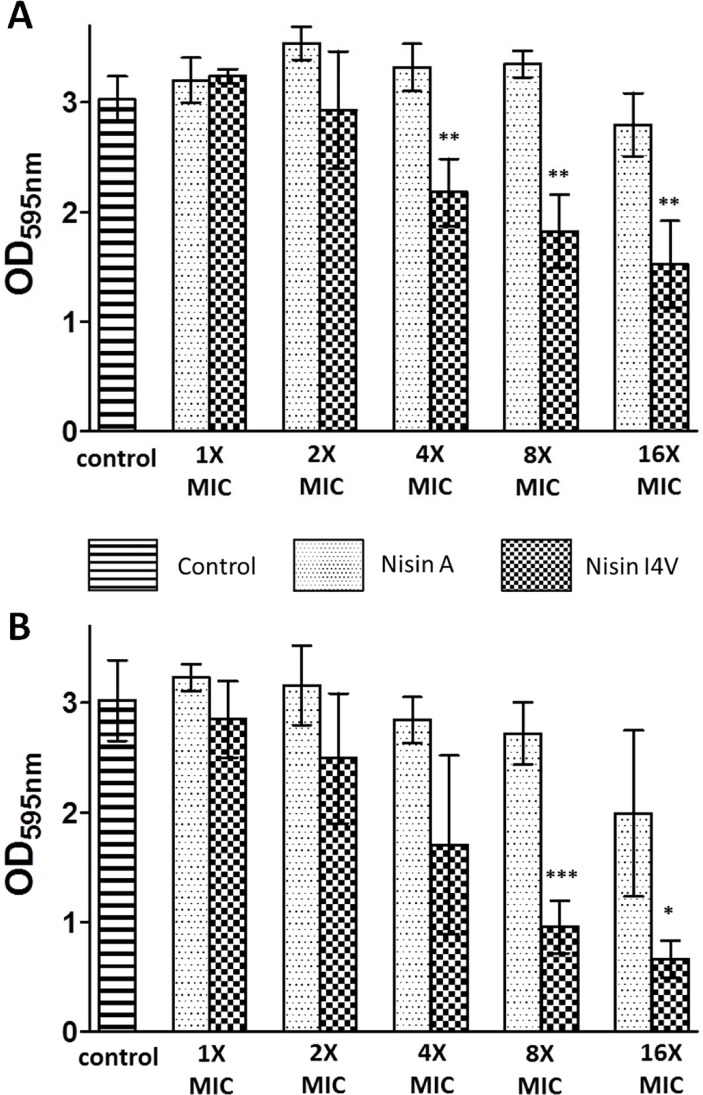
Treatment of biofilms with nisin A and nisin I4V peptides. (A) *S. pseudintermedius* DK729 and (B) *S. pseudintermedius* DSM 21284 with 1, 2, 4, 8 and 16X MIC of nisin A and nisin I4V peptides for 24 hrs as evaluated by crystal violet (CV) staining. The amount of biofilm was quantified by measuring the OD_595_ of CV dissolved in acetic acid. The means and standard deviations of triplicate determinations are presented. Asterisks indicate statistically significant differences (Student’s t-test) between peptides used at similar concentration (* = *p* < 0.05, ** = *p* < 0.01, *** = *p* < 0.001).

Next, biofilms of *S. pseudintermedius* DK729 and *S. pseudintermedius* DSM21284 pre-formed on a 96-well plate were incubated with the peptides at a concentration of 1X, 2X 4X, 8X and 16X MIC for 24 hours. Subsequently, the biofilm mass was determined by crystal violet staining and optical density readings at 595 nm. In the case of *S. pseudintermedius* DK729, a statistically significant reduction (*p* < 0.01) in biofilm mass was observed at 4X, 8X and 16X MIC (1.25, 2.5 and 5 μM, respectively) of I4V treated biofilms compared to untreated control biofilms or biofilms treated with the corresponding concentration of nisin A (Fig. 4A). Similarly, a reduction in biomass was observed for *S. pseudintermedius* DSM21284 biofilms treated with 8X (*p* < 0.001) and 16X (*p* < 0.01) MIC of I4V peptide (equivalent to 2 and 4 mg/L, respectively) compared to the wild-type nisin A treated biofilms (Fig. 4B).

**Fig 5:**
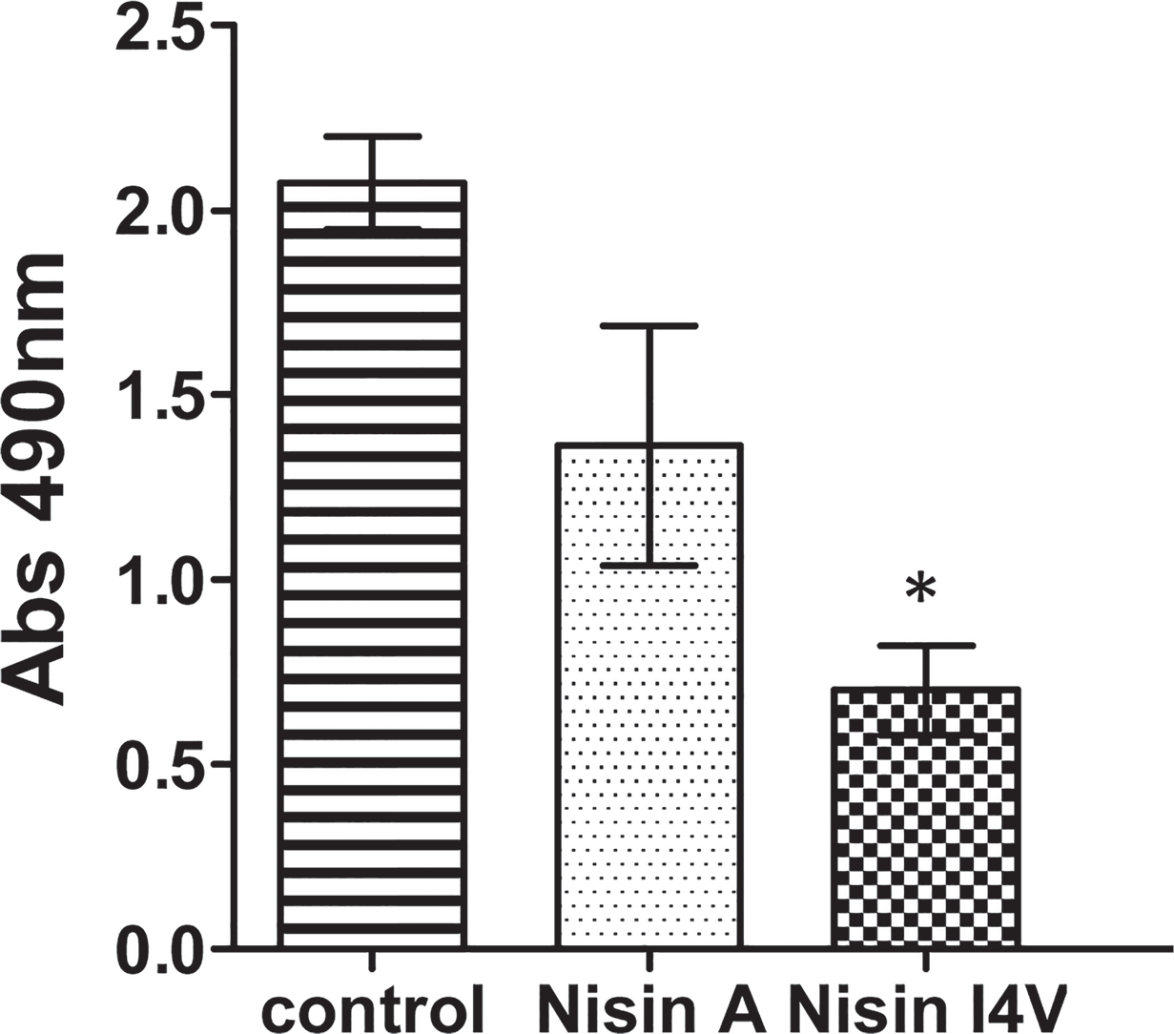
Colorimetric readings of biofilms. Viability of *S. pseudintermedius* DK729 following treatment with 16X MIC of nisin A and nisin I4V peptides and untreated control for 24 hrs as evaluated by the XTT (2,3-bis[2-methyloxy-4-nitro-5-sulfophenyl]-2H-tetrazolium-5-carboxanilide) assay measured using a microtiter plate reader. Asterisks indicate statistically significant differences (Student’s t-test) between peptides used at similar concentration (* = *p* < 0.05).

**Fig 6:**
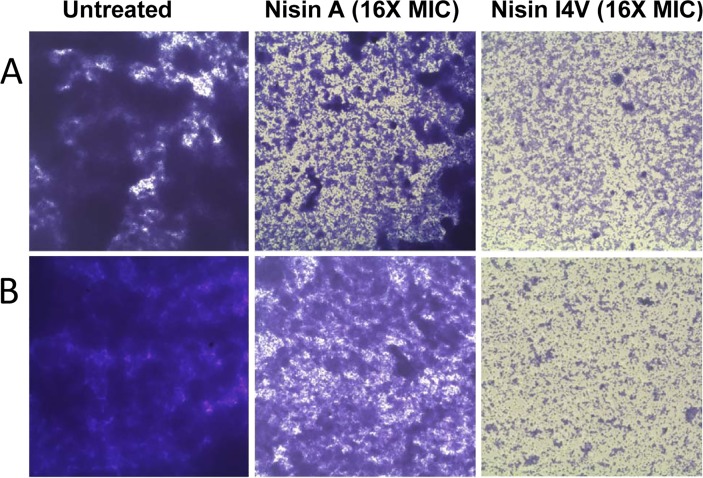
Morphology of nisin-treated biofilms examined by microscopy. (A) Examination of *S. pseudintermedius* DK729 and (B) *S. pseudintermedius* DSM21284 biofilms (magnification 1000X) after 24 hour treatment with 16X MIC of nisin A (Wt) and nisin I4V peptides.

Further analysis was carried out to examine the effect on cell viability of the biofilms following nisin treatment using a rapid colorimetric assay (XTT). The results reveal that following treatment of *S. pseudintermedius* DK729 with 16X MIC nisin A and nisin I4V, viable cells are still present in the biofilm in each case (Fig. 5). The lower absorbance values observed for nisin I4V compared to that for nisin A is likely due to the reduction in biofilm density following peptide treatment (in agreement with previous crystal violet assays (Fig. 6). However, these findings are significant in that the XTT assay provides evidence relating to the viability of the remaining biofilm cells following peptide treatment which cannot be established via crystal violet staining.

## Discussion

*S. pseudintermedius* has emerged as a major challenge for veterinary practitioners owing to its extensive multi-drug resistance and its characteristics as a nosocomial pathogen. Moreover, its ability to form biofilms, complex structures that confer increased resistance to chemotherapies and host defense mechanisms, serves only to compound the problem. As the emergence of MRSP is most likely linked to selective pressure from antimicrobials, more stringent limitations on their use in companion animals may become a reality. Indeed, the prospective use in animals of antimicrobials licensed in human medicine, such as vancomycin, mupirocin and rifampicin is controversial, due to the risk for development of resistance against these agents [45]. Consequently, research into the most advantageous treatment strategies for existing antimicrobial agents as well as alternatives to conventional therapy is urgently required. Due to their many unique properties, the lantibiotic class of bacteriocins would seem to have the potential to breach the gap between effective antibiosis and increasingly drug-resistant clinical and veterinary microbes. Because lantibiotics are produced as gene-encoded pre-peptides, they are much more amenable than classical antibiotics to bioengineering which could lead to the generation of a new arsenal of potent antimicrobials. The identification of bioengineered lantibiotic derivatives with improved activity is becoming a more frequent event as a result of the creation of larger banks of engineered peptides [33,46,47]. Here, we employed a PCR-based bioengineering strategy to create approximately 3,000 nisin derivatives encompassing 19 amino acid positions of nisin not previously targeted by our laboratory with the aim of identifying derivatives with enhanced potency against *S. pseudintermedius*. Although studies relating to the effectiveness of nisin against strains of *S. pseudintermedius* have not been published to date, previous investigations have been carried out on the antimicrobial efficacy of nisin against methicillin resistant strains of staphylococci (N = 100) isolated from companion animals (*S. aureus*, *S. intermedius and S. schleiferi*) (40). The values obtained are in close agreement with the MIC values of nisin A against *S. pseudintermedius* and *S. intermedius* strains as determined in this study (1–3 mg/L). The fact that the activity of nisin I4V against these strains is enhanced is notable given their capacity to become multi-drug resistant. Indeed, antibiotic susceptibility tests carried out against *S. pseudintermedius* DK729 and DSM21284 and *S. intermedius* DSM20373 indicated that these strains are resistant to a variety of antibiotics, including ampicillin, streptomycin, erythromycin and clindamycin (data not shown). The enhanced efficacy of nisin I4V against these targets reveals that the mechanisms *via* which these pathogens have developed resistance to antibiotics do not negate the beneficial consequences of the I4V change. The fact that nisin I4V prevents biofilm formation more successfully than parental nisin A, and indeed is also more effective at reducing the density of established biofilms, is a significant finding. Critically, several studies have shown that nisin can penetrate even the deepest part of a biofilm matrix [22,48]. Davison and co-workers demonstrated that nisin caused a rapid and uniform loss of green fluorescence from all parts of a *Staphylococcus epidermidis* biofilm [48]. Indeed, nisin (MW, 3354) accessed the interior of biofilm cell clusters faster than the other smaller compounds under examination, including a quaternary ammonium compound (MW, 357) and chlorine (MW, 50). The authors proposed that the biofilm penetration time of nisin implies that this agent is not neutralized or bound by the cells or matrix of the biofilm and notably, no evidence of a nisin-tolerant subpopulation was seen [48]. Nisin A has also been shown to be effective against biofilms of several strains of *S. aureus* (including MRSA) and *S. epidermidis* [22]. However, despite showing the effectiveness of nisin to kill biofilm-associated cells, none of these studies reported any significant reduction in biofilm density at the concentration of nisin used. It is thus significant that the nisin I4V peptide exhibits superior activity in reducing biofilm density compared to nisin A and is the first such report for a bioengineered peptide. Future mode of action studies will concentrate on determining the basis of this novel finding.

While the mechanisms underlying the enhanced antimicrobial activity of lantibiotic variants have yet to be elucidated, the fact that lantibiotic properties can be improved is significant. In the case of nisin A, it would appear that ring A is an exceptional target for bioengineering-based approaches to generate more potent microbial inhibitors. Previous mutational studies generated two ring A mutants [where ISL (Ile-Ser-Leu) at positions 4–6 were converted to KSI (Lys-Ser-Ile) or KFI (Lys-Phe-Ile)] that were found to possess superior antimicrobial activity against some non-pathogenic strains, in addition to showing an enhanced capacity to inhibit the outgrowth of spores of *Bacillus subtilis* [32]. Interestingly, two natural nisin variants have been reported that contain substituted residues at the position corresponding to isoleucine 4 in nisin A. Nisin U, produced by strains of *Streptococcus uberis*, has 10 residue substitutions relative to nisin A which include a lysine at position 4 [49]. Recently, a novel nisin variant has been described in which valine replaces isoleucine at position 4 (I4V) and alanine replaces leucine at position 16 (L16A) [50]. Although it exhibits inhibitory activity against a range of staphylococci, *Listeria* and lactococci, the activity of this natural variant compared to its nisin A equivalent has not been determined.

The opportunity also exists to combine nisin I4V with other antimicrobial agents, including naturally derived compounds and currently utilized antibiotics, with a view to identifying synergistic anti-biofilm combinations. From a commercial perspective, it is notable that neither Nisin A nor any other lantibiotic is currently employed commercially as a clinical antimicrobial. Its potential with respect to clinical applications is strengthened by laboratory based experiments highlighting its activity against human pathogens, including multi-drug resistant strains [51,52]. Nisin could be applied in the form of a topical therapy as a treatment for generalized bacterial skin infections, and/or used as an adjunct to systemic therapy. Indeed, combinations of several topical antimicrobial preparations have been successful in the treatment of 6 out of 12 cutaneous MRSP infections [53]. Topical antimicrobial ingredients proven effective in cases of pyoderma include chlorhexidine, benzoyl peroxide, ethyl lactate, mupirocin, fusidic acid and nisin [54]. Alternatively, nisin could also be an effective inhibitor for biofilms which form on in-dwelling devices or hospital equipment.

In conclusion, we have demonstrated the superior capacity of a bioengineered nisin derivative to prevent biofilm formation as well as reduce the density of an established *S. pseudintermedius* biofilm, which may have future applications as a stand-alone treatment or in combination with antibiotics for the elimination of problematic biofilms and associated infections.
